# Effects of Grape Pomace Powder Alone and in Combination with Pomegranate Peel Extract and Lactic Acid to Prolong the Shelf Life of Chicken Nuggets

**DOI:** 10.3390/foods14122040

**Published:** 2025-06-10

**Authors:** Maria Luigia Di Corcia, Adriana Lordi, Federica Moccia, Amalia Conte, Matteo Alessandro Del Nobile

**Affiliations:** 1 Department of Humanistic Studies, Letters, Cultural Heritage, Educational Sciences, University of Foggia, Via Arpi, 176, 71121 Foggia, Italy; maria.dicorcia@unifg.it; 2 Department of Economics, Management and Territory, University of Foggia, Via A. da Zara, 11, 71122 Foggia, Italy; adriana.lordi@unifg.it (A.L.); matteo.delnobile@unifg.it (M.A.D.N.); 3 Department of Chemical Sciences, University of Naples, Via Cintia, 4, 80126 Naples, Italy; federica.moccia@unina.it

**Keywords:** grape pomace, lactic acid, pomegranate peel extract, chicken nuggets, shelf life, by-products

## Abstract

In this study different strategies have been adopted to promote the shelf-life prolongation of fresh chicken nuggets. Two different by-products, grape pomace powder (GPP) and pomegranate peel extract (PE), alone and in combination with lactic acid, were suggested as meat preservatives. The antioxidant properties measured by different assays confirmed the presence of bioactive compounds in both by-products. To test their effects on meat samples, a control nugget without any compounds and a nugget with sole lactic acid were also used for comparison. During a refrigerated storage of about 2 weeks, microbiological stability and changes in sensory properties were monitored to assess the product shelf life. Results demonstrated that GPP promoted a good preservation of meat, and its combination with other compounds further increased the effectiveness. The control without any active agent remained acceptable for less than 1 day, the control with lactic acid for less than 2 days (*p* > 0.05). The other active samples lasted longer depending on the combinations of active compounds. When the sole GPP was used, about 3 days of shelf life were recorded (*p* < 0.05). While the combination of GPP with PE promoted only a slight increase of shelf life by 1 day (2.9 vs. 3.9 days), the combination of GPP with LA prolonged the shelf life to more than 6 days (2.9 vs. 6.58 days) (*p* < 0.05). The best results were found when both LA and PE were combined with GPP because the nuggets lasted about 10 days (*p* < 0.05). To better highlight the single and combined effects of the tested active agents, a proper index $\left(∆\%\right),$ comprising the normalized percent difference between the shelf life of the sample with the most antimicrobials and that of the sample with the least antimicrobials, was calculated, thus comparing the various samples and demonstrating the potential synergic effects among them.

## 1. Introduction

Poultry meat is a very popular food, due to its low production costs, low fat content and high nutritional value. However, poultry meat products are very perishable and require particular attention during preparation, storage and distribution to guarantee a desired shelf-life [1]. Fresh chicken breast has a short storability (3–5 days) due to its high water content, poly-unsaturated fatty acid that can provoke rancidity, and very pronounced tendency towards microbial contamination [2]. To prolong shelf-life, various approaches have been used over the years, such as the addition of spice extracts to delay microbial growth and lipid oxidation [3], the application of chitosan films with natural antioxidants of peanut husk extracts and pink pepper residues [4], combined treatments of high and moderate pressure with the addition of antimicrobial edible coating [5], oxygen absorber, and citrus extract [6].

Among the products that are based on fresh chicken, chicken nuggets play a key role, mainly due to their convenience. Current hectic lifestyles cause changes in consumers’ eating habits, highlighting an increase in demand for so-called ready-to-cook products [7]. The main components of chicken nuggets are represented by chicken meat, which is breaded with batter, and frying, which is used to improve the quality and organoleptic characteristics in terms of flavor, appearance, crunchiness and consistency [8]. To prolong the shelf-life of chicken nuggets, some authors have proposed the replacement of wheat flour, used for the batter, with quinoa flour, to delay oxidation of lipids and proteins, and prevent microbial contamination during cold storage [9]. Another work has shown how the phenolic compounds added as natural extracts of seeds, herbs and fruits, together with organic forms of Zn and Se, can delay microbial growth, reduce protein and lipid oxidation and maintain the sensory quality of chicken nuggets [10]. Raeisi et al. [7] have proposed co-encapsulated fish oil and garlic essential oil to retard chemical deterioration and microbial proliferation, also improving the sensory properties and shelf life of samples during refrigeration.

The growing awareness of consumers regarding the food–health relationship has increased the need to preserve food products using natural preservatives and apply environmentally friendly technologies [11], giving more attention to sustainability [12]. In fact, in recent years, several researchers have focused their attention on by-products from fruits and vegetables as new food ingredients [13,14]. Worldwide, it is estimated that the agri-food industry produces more than 190 million tons of by-products each year [15]. By-products derive from primary food production processes, such as juice/nectar processing, wine production, and olive oil extraction [16], but also includes agricultural waste from the field. By-products of the agri-food sector can differ from produce and vary according to the morphological parts, they can include portions of fruits and vegetables (leaves, peel, skin, seed, etc.) or can include products that are obtained because of morphological defects, the absence of appropriate handling processes or which have remained rejected for various reasons [17]. In this context, the recycling of fruit and vegetable by-products represents a valid opportunity to increase food sustainability and to ensure sustainable consumption and production patterns, while also considering that by-products are reported to have high nutritional value to be used as functional ingredient [18]. Generally, these residues constitute a good natural source of carbohydrates, polysaccharides and bioactive molecules, such as proteins, vitamins, minerals and antioxidants [19]. Among by-products, grape pomace has been abundantly studied and is highly appreciated for being rich in many bioactive compounds, such as catechin, epicatechin and tannins, which confer high antioxidant and antimicrobial power and promote shelf-life preservation when applied to food [20]. Another interesting by-product is pomegranate peel. The recognized antioxidant, antimicrobial and antifungal properties are due to the presence of tannins and other phenolic compounds that allow the successful application of pomegranate peels to numerous food applications [21].

In this study, for the first time, grape pomace and pomegranate peel extract, in combination with lactic acid, were used to substitute the traditional batter of fresh chicken nuggets. The study aimed to propose a new approach for poultry meat preservation, based on by-product recycling, so as to be more sustainable and easily scalable. To this aim, different formulations were realized and tested during storage for sensory acceptance and microbiological stability. A global quality index was then calculated using the shelf life values, to evaluate single and combined effects of tested ingredients.

## 2. Materials and Methods

### 2.1. Grape Pomace Powder (GPP) Preparation

Grape pomace from *Vitis vinifera* (cv. Montepulciano) was kindly provided by the local farm Cantine della Bardulia (Barletta, Italy) as a by-product after wine production. The pomace contained around 85% moisture content. To avoid enzymatic degradation and contamination, the by-product was immediately stored at −18 °C. After defrosting under refrigerated temperature, a hot air drying was carried out in a conventional dryer (PF-SICCO80PRO, SICCO TECH, Campobasso, Italy) using forced convection at atmospheric pressure, setting the temperature at 60 °C and the relative humidity at 5% [22]. Drying data were obtained by periodic weighing of the samples (5 g) using a thermal balance (MA 150Q, Sartorius, Gottingen, Germany) set at 130 °C. Drying was completed when the weight was constant for various consecutive measurements.

### 2.2. Pomegranate Peel Extract (PE) Preparation

Pomegranate peel powder (230 g), obtained after juice preparation by squeezing followed by lyophilization, was extracted with an ethanol/water solution (6:4, *v*/*v*) at a solid-to-liquid ratio of 100 mg/mL. The mixture was stirred for 60 min at room temperature and subsequently centrifuged at 7000 rpm for 10 min at 4 °C. The resulting supernatant was collected and stored at −25 °C, while the solid residue was lyophilized to determine the extraction yield by weight difference (57% *w*/*w*). Before use, PE was concentrated under reduced pressure using a rotary evaporator until complete removal of ethanol.

### 2.3. Evaluation of the Antioxidant Properties of GPP and PE

The antioxidant properties of GPP and PE were evaluated by the 2,2-diphenyl-1-picrylhydrazyl (DPPH) assay, the ferric reducing/antioxidant power (FRAP) assay, the cupric ion reducing antioxidant capacity assay (CUPRAC) and total phenolic content (TPC). For the DPPH assay, PE and GPP were added to a 0.2 mM ethanolic solution of DPPH (final dose 0.0133–0.8 mg/mL) and, after 10 min under stirring at room temperature, the absorbance of the solution at 515 nm was measured. Trolox was used as a reference antioxidant. Experiments were run in triplicate [23,24]. For the FRAP assay, PE and GPP were added to 0.3 M acetate buffer (pH 3.6) containing 1.7 mM FeCl3 and 0.83 mM TPTZ, (final dose 0.0013–0.2 mg/mL) and, after 10 min under stirring at room temperature, the absorbance of the solution at 593 nm was measured. Results were expressed as Trolox equivalents (eqs). Experiments were run in triplicate [25]. For the CUPRAC assay, 1 mL of a 10 mM CuCl_2_ solution in water, 1 mL of a 7.5 mM neocuproine solution in ethanol, 1 mL of 1 M ammonium acetate buffer (pH 7), and 900 μL of distilled were mixed and PE and GPP were subsequently added (final dose 0.0027–0.2). The mixtures were stirred for 30 min at room temperature. After incubation, the absorbance was measured at 450 nm. Results were expressed as Trolox equivalents (eqs). Experiments were run in triplicate [26]. For the TPC assay, GPP and PE were added at a final dose of 0.0133–0.5 mg/mL to a solution consisting of Folin–Ciocalteu reagent, 75 g/L Na_2_CO_3_, and water in a 1:3:14 *v*/*v*/*v* ratio. After 1 min incubation at room temperature, the absorbance at 765 nm was measured. Gallic acid was used as reference compound. Experiments were run in triplicate [27].

### 2.4. Sample Preparation

The ingredients for preparing the breaded chicken nuggets were wheat flour, breadcrumb, sparkling water, and skinless chicken breast, all purchased from a local market (Foggia, Italy). In the first step, three types of batter were prepared as described in Table 1. The control batter had no by-products (#A), one batter contained pomace powder (#B), and the other one contained both pomace powder and pomegranate extract (#C), in substitution of part of the wheat flour.

The chicken breast was cut into bite-sized pieces, each about 2 cm thick and weighing approximately 10 g. Depending on the batter selected among (#A), (#B) and (#C), on the use of a previous dipping in lactic acid (4% solution, Greensistem, Foggia, Italia) and depending on the type of final coating to bread the samples (breadcrumb or GPP), a total of six samples were prepared: two controls and four active samples. The control samples (CTRL 1 and CTRL 2) were obtained by dipping the meat pieces into batter #A, followed by coating in traditional breadcrumb. For CTRL 2, these steps were preceded by an additional dipping in a 4% lactic acid solution. The active samples were prepared using the active batters (#B and #C). Specifically, the first sample (SAMP_1) was prepared by dipping the chicken into batter #B, followed by coating in the GPP. The second sample (SAMP_2) was dipped in 4% lactic acid, then in batter #B and finally coated with GPP. Another sample (SAMP_3) followed the same preparation procedure as SAMP_1, but using active batter #C instead of #B. The last sample (SAMP_4) was prepared in the same manner as SAMP_2 but adding a previous dipping in a 4% lactic acid solution. All of the formulations are summarized in Table 2.

All of the concentrations adopted in the current study for each active ingredient (LA, GPP and PE) come from a proper preliminary screening carried out in the lab, based on both antimicrobial efficacy and sensory acceptance, whose data are not published.

After the preparation, two nugget samples of each typology were placed on a food-grade pad and packaged in air using a multi-layer bag (nylon/polyethylene), provided by Myvac (Bolzano, Italy), and stored in the refrigerator (4 ± 1 °C) for about two weeks. All samples were examined on days 0, 1, 2, 3, 6, 7, 8, 9, 10, and 13 to monitor microbiological quality, pH, and sensory properties.

### 2.5. Microbiological Analyses

During the entire storage period, both control and active samples were analyzed for microbial quality. All samples were aseptically weighed into sterile Stomacher bags, diluted with peptone water (dilution 1:10) and homogenized for 120 s with a Stomacher LAB Blender 400 (Pbi International, Milan, Italy). Subsequently, decimal dilutions of the homogenized samples were made using the same diluent, and the dilutions were plated on to specific media in Petri dishes to enumerate the following microbial groups: Plate count agar (PCA, Oxoid) incubated at 30 °C for 48 h for mesophilic bacteria and 5 °C for 10 d for psychrothrophic bacteria; pseudomonas agar base (PAB, Oxoid), with an added cetrimide fucidin cephaloridine (CFC) selective supplement, incubated at 25 °C for 48 h for *Pseudomonas* spp.; violet red bile glucose agar (VRBGA, Oxoid) incubated at 37 °C for 24 h for Enterobacteriaceae; Baird–Parker agar, supplemented with egg yolk tellurite emulsion, incubated at 37 °C for 48 h, was used for *Staphylococcus* spp.; finally, coco-shaped lactic acid bacteria were counted on de Man, Rogosa and Sharpe agar containing cycloheximide (0.1 g/L; Sigma-Aldrich; St. Louis, MO, USA) after incubation at 37 °C for 48 h. At each sampling time, two different samples were used for test repetition. Microbial thresholds were set to 10^7^ CFU/g for total viable mesophilic bacteria, *Pseudomonas* spp. and lactic acid bacteria; 10^4^ CFU/g for *Staphylococcus* spp.; and 10^6^ CFU/g for Enterobacteriaceae [28].

### 2.6. Sensory Analysis

The sensory quality of chicken nugget samples was assessed by seven trained panelists with ages of between 27 and 50 years, who were researchers and PhD students of the food department of the University of Foggia, and who had previous experience in the sensory analysis of meat-related products. Though they were expert at sensory evaluation, new training sessions were carried out with reference to nuggets. The members met in two sessions (1.5 h/day) to select adequate sensory parameters, identify a proper scale, and to align their judgements using commercially available nuggets. During the test, at each sampling time, the panelists were asked to indicate the following attributes of both cooked and uncooked samples: color, odor, appearance, and texture of uncooked samples and color, odor, appearance, texture, crunchiness, and sandiness of cooked samples. To cook the products, the chicken nuggets were fried in hot sunflower oil at 180 °C for 90 s. Moreover, each panel member was asked to give the global sensory score of cooked and uncooked samples. A 9-point scale was used for the evaluation of each specific attribute and of the global sensory quality (1 = lowest score; 9 = highest score), where a score of 5 indicates the limit at which the quality of the product was found unacceptable. A protocol was adopted to protect the rights and privacy of each panelist. This protocol required the verbal consent of the participants to participate, without any coercion and with the ability to withdraw from the study at any time. The panelists were fully aware of the study requirements and risks, and no data were released without their knowledge.

### 2.7. Measurements of pH

The pH measurement was performed on the first homogenized dilution of each sample, using a pH meter (Crison, Barcelona, Spain). At each sampling time the pH was measured on two different samples.

### 2.8. Shelf Life Calculation

According to other literature references in which the shelf life of fresh meat was calculated on the basis of microbiological and sensory quality evolution [29,30], the current work used a modified version of the Gompertz equation to calculate the microbiological acceptability limit (MAL) and the sensory acceptability limit (SAL), by fitting both microbiological and sensory experimental data. The lowest value among the calculated MAL and SAL values was identified as the product shelf life, being the time in days in which the product maintained an acceptable quality.

The following index was used to assess the single and combined effects of tested antimicrobials on the shelf life of chicken nuggets:

(1) ∆% = (SL_A_ − SL_B_)/SL_B_∙100

where (SL_A_) is the shelf life of the sample with the most antimicrobials and (SL_B_) is the shelf life of the sample with the least antimicrobials. In fact, ∆% is the normalized percent difference between the shelf life of the sample with the most antimicrobials, which is supposed to have a longer shelf life, and that of the sample with the least antimicrobials, which is supposed to have a shorter shelf life. If ∆% > 1, there is an effect of the used antimicrobials on the shelf life (i.e., the used antimicrobials extend the food’s shelf life). The contrary is true when ∆% ≤ 1.

### 2.9. Statistical Analysis

Experimental data are reported as mean ± standard deviation (2 replicates). Both experimental data and fitting parameters were compared by a one-way analysis of variance (HSD of Tukey), with the option of homogeneous groups (*p* < 0.05), to determine significant differences among samples. JMP 18 for Windows (JMP Statistical Discovery LLC 920 SAS Campus Drive Cary, NC 27513, USA) was used.

## 3. Results and Discussion

### 3.1. Antioxidant Properties of GPP and PE

At first, the antioxidant properties of GPP and PE were investigated by widely used assays, i.e., DPPH, FRAP, CUPRAC, and TPC assays. In the case of GPP, the experiments were carried out following the “QUENCHER” method, which allows one to measure the efficiency of electron transfer processes from a solid antioxidant [23,24,25,26,27]. As shown in Table 3, both samples exhibited remarkable antioxidant properties, which were significantly higher when compared with other agri-food by-products, such as spent coffee grounds, banana, pineapple, apple, and orange by-products [31,32]. The high antioxidant activity observed is attributed to the presence of significant amounts of phenolic compounds in both by-products. In particular, pomegranate peels are rich in hydrolysable ellagitannins (mainly punicalagin and punicalin), endowed with very high antioxidant potency. On the other hand, grape pomace is rich in condensed tannins, together with small phenolic acids and anthocyanins [31]. Notably, PE exhibited stronger antioxidant properties and TPC values that were about 5-fold higher than those of the GPP sample. This difference in antioxidant activity and polyphenol content could be due to the different physical states of the two samples. In the case of GPP, the solid nature could decrease the solubility and dispersion of the sample in the assay medium, reducing the efficiency of electron or hydrogen transfer processes, leading to a decrease in the measured values.

### 3.2. Chicken Nugget Shelf Life

To assess the product shelf life, microbiological quality, pH and sensory properties were monitored during about two weeks of storage at 4 °C.

**Figure 1:**
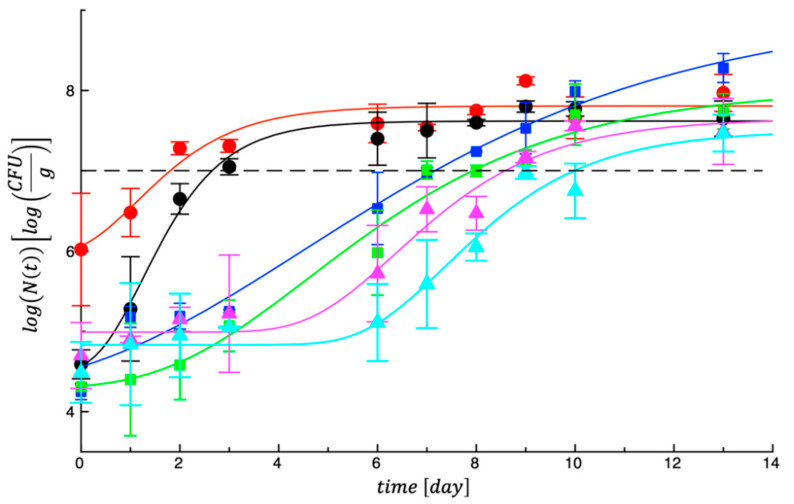
Evolution of total mesophilic bacteria in controls and treated meat samples during storage at 4 °C. 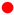 CTRL_1; 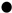 CTRL_2; 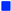 Samp_1; 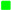 Samp_2; 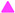 Samp_3; 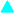 Samp_4. The curves are the best fit to the experimental data. 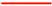 CTRL_1; 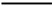 CTRL_2; 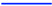 Samp_1; 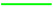 Samp_2; 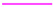 Samp_3; 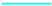 Samp_4. The dotted line represents the threshold for acceptability (10^7^ cfu/g).

As regards the evolution of spoilage microorganisms, Figure 1 reports a trend of the experimental data of mesophilic bacteria recorded in our samples. As can be observed in the figure, total mesophilic counts in the two control samples grew rapidly and overlapped the microbial threshold within the first 3 days of storage. On the other hand, in the same figure it can be observed that the other four treated samples recorded a different microbial evolution, with growth rate more delayed and lag phase more prolonged, if compared with those of the control meat. To better highlight differences among samples, a fitting procedure was adopted, and the MAL value was calculated for each specific microbial specie, as main fitting parameter.

In Table 4 are listed the MAL values recorded for the mesophilic bacteria. As can be seen, the first column of Table 4 reports statistically significant different (*p* < 0.05) values among tested samples. In particular, it is worth noting that low values around 2 days were recorded for both the control nuggets, whereas MAL around 7, 8 and 9 days were calculated, depending on the combination of GPP with LA, PE or both, respectively. The effects are not surprising considering the well-known properties of these active agents, as assessed from data reported in Table 3. Literature on grape pomace applied as breading to meat products is not abundant but much information is available on extracts from grape pomace and grape seeds applied to raw and cooked beef, pork and chicken meat, with interesting effects on microbial proliferation and antioxidant activity.

Though environmental and agricultural factors can influence the composition of grape extracts, microorganism numbers generally decreased in proportion to extract concentration [33,34]. The use of pomegranate by-products as a source of natural antioxidant and antimicrobial compounds in processed meat products has been also investigated and the effects on microbial growth have been abundantly demonstrated [35]. Kannat et al. [36] enhanced the shelf life of chicken meat products by 2–3 weeks during chilled storage with pomegranate peel extract. They found that pomegranate seed extract did not have any significant activity, while pomegranate peel extract showed excellent antioxidant potential, good reducing power and iron chelation capacity and good antimicrobial activity.

**Figure 2:**
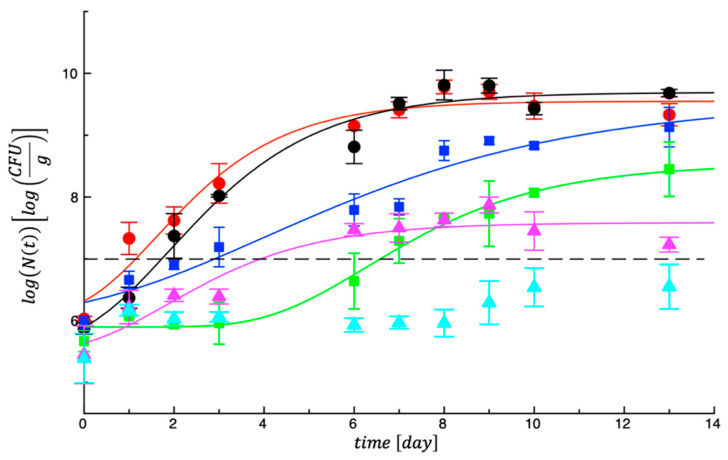
Evolution of *Pseudomonas* spp. in controls and treated meat samples during storage at 4 °C. 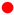 CTRL_1; 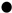 CTRL_2; 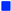 Samp_1; 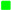 Samp_2; 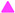 Samp_3; 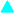 Samp_4. The curves are the best fit to the experimental data. 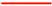 CTRL_1; 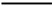 CTRL_2; 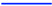 Samp_1; 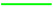 Samp_2; 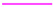 Samp_3; 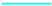 Samp_4. The dotted line represents the threshold for acceptability (10^7^ cfu/g).

As regards *Pseudomonas* spp., Figure 2 reports trends in the data. In this figure it is notable that the two control samples grew in a few hours from the day of production and packaging, whereas the other four samples present different microbial evolutions. In particular, microbial growth was more delayed for the nuggets where GPP was combined with LA (Samp_2) and where GPP was combined with LA and PE (Samp_4).

These data trends report an important lag phase and Samp_4 in particular maintained a very low bacterial count for the entire observation period. In Table 4 the calculated MAL values for *Pseudomonas* spp. can be found. Compared with the control meat, the other samples recorded higher values, though the two best antimicrobial solutions were found with Samp_2 and Samp_4. This result can be ascribed to the effects of LA, which acts in a synergistic way when combined with other active agents. In fact, data from literature confirm the effects of bioactive compounds and organic acids applied to a wide range of foods against different types of spoilage and foodborne pathogens [37].

**Figure 3:**
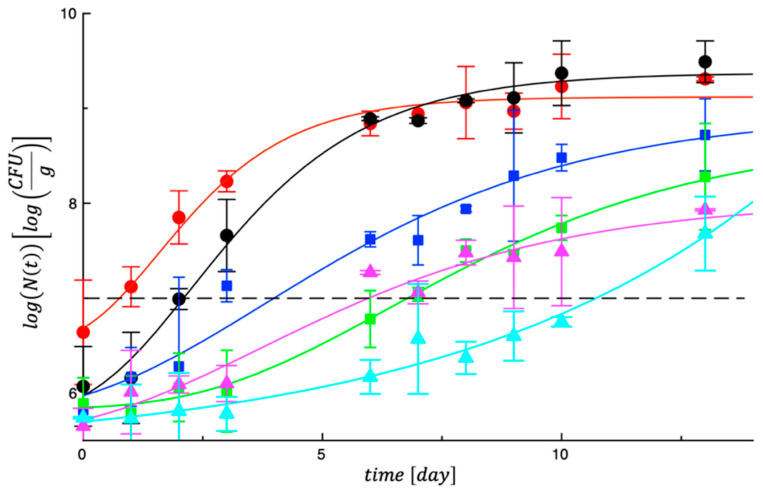
Evolution of total psychrotrophic bacteria in controls and treated meat samples during storage at 4 °C. 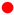 CTRL_1; 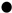 CTRL_2; 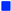 Samp_1; 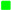 Samp_2; 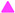 Samp_3; 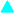 Samp_4. The curves are the best fit to the experimental data. 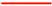 CTRL_1; 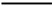 CTRL_2; 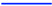 Samp_1; 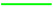 Samp_2; 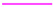 Samp_3; 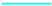 Samp_4. The dotted line represents the threshold for acceptability (10^7^ cfu/g).

Figure 3 reports cell counts of psychrothrophic bacteria. Psychrotrophic bacteria are the most prevalent bacteria on refrigerated chicken meat, and are the microorganisms of choice to detect the true microbial loads of chicken products [38]. As can be noted, very similar trends to mesophilic bacteria were found also for these spoilage microorganisms. In both control products the bacteria grew in little time, whereas a significant delay was observed for the other four samples. The data in Table 4 for psychrothrophic bacteria confirm these trends and show evidence that the first control became unacceptable within the first day of storage. About 2 days are necessary for the second control where lactic acid was used to treat meat. When GPP was used, the time to reach the microbial threshold significantly increased up to about 4 days (*p* < 0.05). The best combinations are represented by GPP combined with LA (about 7 days) or with both LA and PE (about 10 days). Literature findings about the effects of these antimicrobials on mesophilic bacteria also support this experimental evidence. Rodríguez-Melcón et al. [39] compared nisin, sodium nitrite, and lactic acid on the prevalence and antibiotic resistance patterns of the *Listeria monocytogenes* that is naturally present in poultry and preliminarily verified the effects of these agents on the levels of certain microorganisms that indicate hygiene quality (aerobic plate counts, psychrotrophic microorganisms and enterobacteria). They found that, of the three additives studied, lactic acid showed the most effectiveness as an antimicrobial agent. Under the conditions tested, lactic acid alone was able to reduce levels of aerobic plate counts, psychrotrophic microorganisms and enterobacteria when compared with the untreated samples.

During the entire storage period, the *Staphylococcus* spp. was not found in tested samples. For this reason, the experimental data were not fitted and, in the last column of Table 4, the MAL parameter was reported higher than 13 days, as a maximum monitoring time.

Enterobacteriaceae were only found in both the control samples. This means that the use of single GPP or its combination with other agents was effective in preventing the enterobacteria proliferation. García-Lomillo et al. [40] have also found that products derived from grape pomace completely inhibit Enterobacteriaceae growth. Therefore, Table 4 only reports MAL values for the two controls and lists an MAL higher than 13 days for the other four meat products.

The lactic acid bacteria grew very slowly and reached counts of around 10^6^ CFU/g after 9–10 days in all of the samples (data not shown). Only in two cases, Samp_1 and Samp_2, was a slight overlapping of the threshold set to 10^7^ CFU/g recorded (see Table 4). The effects on the growth of different meat spoiling microorganisms, such as lactic acid bacteria, depend on polyphenol concentration, medium, and species [33]. Various studies have suggested that polyphenols may improve sugar metabolism in lactic acid bacteria, thereby stimulating proliferation [41,42].

The pH of meat samples did not remain constant over time for any of the samples (data not shown), thus suggesting possible correlation with microbial growth. In the two control meat products a reduction was observed from about 6.7 to about 5.6, without any statistically significant differences among them (*p* > 0.05). This recorded pH reduction is in line with other literature evidence [9]. When sole GPP was used, the pH started from about 5.2 and reached about 5.6 after 13 days of storage. When GPP was combined with LA (Samp_2) or was combined with PE (Samp_3) or was combined with LA and PE (Samp_4), it maintained values around 5.2 during the entire observation period. Therefore, the low pH of the active samples can be considered an inhibitory factor limiting the growth of bacteria [43]. Further study is still necessary to assess if the direct bactericidal action of tested active ingredients results from a pH decrease within bacterial cells.

Meat samples were also judged for sensory properties, before and after cooking. As regards the sensory quality of uncooked samples (data not shown), it is worth considering that a net difference was found in the evolution of the two control samples compared with the other four products, in good agreement with microbiological data. In fact, both controls became unacceptable after about one week of storage, regardless of the presence of lactic acid. These products were compromised in odor, color, appearance and texture, thus allowing for the recording of an overall quality below the minimum score for acceptability (score = 5) after about 7 days. Meat samples with preservatives recorded a prolonged acceptability which slightly increased considering GPP alone, GPP with LA, GPP with PE and GPP with LA and PE. Data in Table 5 report the results of the fitting procedure carried out on sensory data. As can be seen in the table, an SAL of about 7 days was recorded for the two controls and higher values ranging between 10 and 11 days were calculated for the other samples.

**Figure 4:**
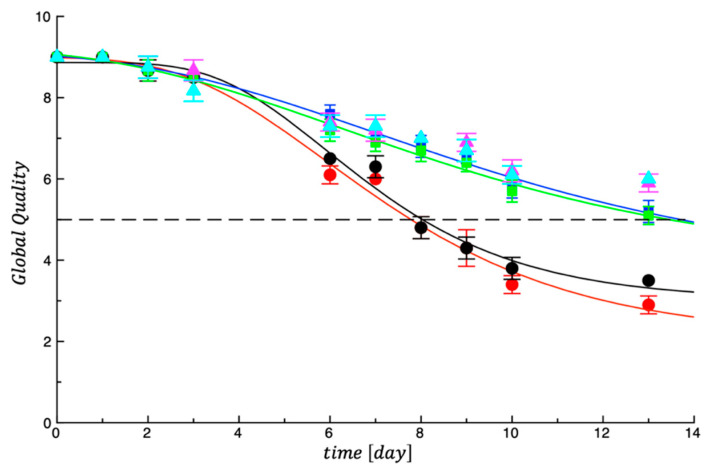
Evolution of global quality of cooked controls and treated meat samples during storage at 4 °C. 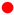 CTRL_1; 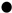 CTRL_2; 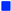 Samp_1; 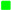 Samp_2; 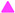 Samp_3; 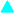 Samp_4. The curves are the best fit to the experimental data. 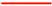 CTRL_1; 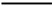 CTRL_2; 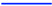 Samp_1; 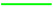 Samp_2; 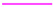 Samp_3; 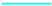 Samp_4. The dotted line represents the threshold for acceptability (score = 5).

Figure 4 reports the data recorded on cooked samples. As one would expect, sensory properties decreased over time because the detrimental phenomena occurring in the meat also generally affect the parameters that are responsible for product acceptability after cooking [7]. A similar trend to that recorded in the uncooked samples was also found for the cooked products.

As can be seen, while the meat of the two control products lost color, odor, appearance and proper texture properties after about 8 days, the other treated samples remained acceptable for more time and, in most cases, never reached any threshold (score = 5). Table 4 also lists the results of the fitting procedure for cooked products. As can be inferred, the two controls recorded SAL values of around 8, Samp_1 and Samp_2 of around 13 days and the last two samples recorded SAL values higher than 13 days. The results of sensory evaluation agree with microbial analyses and are also consistent with previous research where good correlations among sensory attributes and microbial quality were reported [7].

Comparing values of MAL and SAL, it is possible to infer the meat shelf life as the lowest value among them. Considering that the lowest values of acceptability were always recorded for microbiological quality, the shelf life of samples tested in the current study can be inferred from the data in Table 4 (Table 6). The first control product lasts less than one day for an unacceptable proliferation of psychrotrophic bacteria, whereas the control meat treated solely with LA lasts a slightly longer time due to the *Pseudomonas* spp. proliferation. The same pseudomonads group is responsible for the shelf life of Samp_1, Samp_2 and Samp_3, whereas Samp_4 became unacceptable for its high growth of mesophilic bacteria. Compared with the controls, the sample treated solely with GPP recorded a very small shelf-life increase, which became more interesting when PE (3.93 ± 0.70 days) was also added as active agent. The effects on shelf life became more marked when GPP was used in combination with LA (6.58 ± 0.23 days) and when GPP was combined with both LA and PE (9.98 ± 0.54 days) (Table 6).

### 3.3. Effects of Antimicrobials on Shelf Life

To assess the effect of the active ingredient used alone and in proper combination with other compounds, the shelf-life values were used to calculate a normalized percent difference (∆%). To demonstrate the effects of LA on the shelf life, samples CTRL and CTRL_LA were used to calculate the first normalized percent difference, which will be referred to as ∆%_1. According to the shelf-life values recorded for the above sample, a ∆%_1=127 was obtained. The data of the shelf life of the two controls are not statistically different (Table 4) and, therefore, the calculated index, despite the difference recorded between the two samples, is not statistically significant. As can be inferred from the data listed in Table 3, the microbial quality of the investigated samples is the factor that limits the shelf life. Therefore, the fact that ∆%_1≫1 clearly indicates that LA extended the shelf life of chicken nuggets by slowing down the microbial growth of the main spoilage microorganisms. In fact, lactic acid is known to be effective against microbial proliferation in poultry meat [39]. The effect of GPP on chicken nugget shelf life was assessed by comparing the CTRL and Samp_GPP samples. The calculated normalized percent difference between the two mentioned samples (∆%_2) is equal to 281.25. This result indicates that GPP is very effective in extending the shelf life of the investigated food. This is not surprising considering the abundant findings from literature dealing with pomace and its extracts applied to improve the safety and quality of meat products. It is worth noting that ∆%_2 is double ∆%_1, thus suggesting that GPP can be considered more effective than LA in extending the shelf life of chicken nuggets. Direct comparisons between LA and GPP on chicken nuggets from literature evidence are not possible because no studies have been found with similar treatments. However, there are several abilities that are attributed to the phenolic compounds generally contained in grape by-products that indicate a significant effectiveness against microbial proliferation when compared with organic acids. The mechanisms of action of these abilities are associated with the ability to inhibit cell wall synthesis and produce cell membrane alterations with consequent loss of crucial intracellular material, ability to chelate essential metals, to bind polysaccharides, and to proteins, thereby producing compounds that cannot be metabolized by microorganisms and which have the ability to bind vital components such as enzymes and cell transport proteins [40]. When comparing sample CTRL_LA and sample Samp_LA_GPP, an effect of GPP on the sample treated with LA was observed. In this case, the normalized percent difference (∆%_3) is equal to 280.60. As one would expect, the value of ∆%_3 is very similar to ∆%_2, thus proving once again that GPP strongly influences chicken nugget shelf life, due to the well-known properties of grape pomace on meat [33,34]. To assess the effect of PE on microbial growth of our meat-based products, samples SAMP_GPP_PE and Samp_GPP were used to calculate the normalized percent difference (∆%_4). In this case a value of 35.17 was obtained. The value of ∆%_4>1 means that also PE can exert positive effects on nuggets shelf life and it does not surprise if we consider the recognized activity of pomegranate extract on meat [44,45]. The effect of PE on the shelf life of chicken nuggets can be also assessed by using the Samp_LA_GPP and SAMP_LA_GPP_PE samples, thus calculating a new normalized percentage difference (∆%_5). The obtained value is 51.56, which is slighter greater than ∆%_4. The reason for this results is that ∆%_4 measures the effect of PE in presence of GPP, whereas ∆%_5 measures the effect of PE in presence of GPP and LA [46]. The observed difference can be ascribed to the presence of LA which acted synergically with GPP and PE to slow down the microbial quality decay of the investigated food [37]. By comparing the shelf life of the SAMP_LA_GPP_PE and CTRL samples, it is possible to measure the synergy of the three studied antimicrobial agents acting together. In this case the calculated normalized percentage difference (∆%_6) is equal to 1209.50, which is quite high. An attempt was made to evaluate whether there is a synergistic effect between the antimicrobials studied. To this aim, to calculate the normalized percentage increase it was necessary to add the shelf life increase individually obtained by using LA, PE, and GPP, and compare it with shelf life of CTRL. In particular, the CTRL and CTRL_LA samples were used to calculate the sole effect of LA, the CTRL and Samp_GPP samples to calculate the sole effect of GPP, and the Samp_LA_GPP and Samp_LA_GPP_PE samples to calculate the sole effect of PE. The above effects were added together and used to calculate the normalized percentage increase (∆%_7), the value obtained is 853.77. It is worth noting that ∆%_7 does not consider any synergistic effect that the antimicrobials investigated could exercise, it simply adds the effects that individual antimicrobials can have on shelf life. As reported beforehand, ∆%_7 is way lower than ∆%_6. This is not surprising, as in the literature it was reported that antimicrobials can act synergically in slowing down the microbial growth [3,47]. Therefore, the ∆% values are useful to make comparisons and underline differences among samples. Further research could be carried out to better understand the mechanisms of action and the interactions between polyphenols and organic acid that justify the recorded effects in poultry meat.

## 4. Conclusions

In this study the shelf life of chicken nuggets was assessed during two weeks of refrigerated storage. The samples were prepared using different active ingredients used alone and in proper combination. Two controls were also prepared. Microbiological proliferation of main spoilage microorganisms, pH and sensory quality changes of both raw and cooked samples were monitored to assess the day within which the quality remained acceptable. The results demonstrate that all of the samples, regardless of the preservation ingredient adopted, remained acceptable for sensory properties over a longer period of time when compared with microbiological stability and that therefore the product shelf life is strictly linked to the values of the microbial acceptability limit (MAL), calculated for each spoilage group that reached the microbial threshold. For the unacceptable proliferation of psychrotrophic bacteria, the control sample lasted less than 1 day, whereas the undesired proliferation of *Pseudomonas* spp. provoked the end of the shelf life for most of the samples, nuggets with LA (1.730 days), nuggets with GPP (2.905 days), nuggets with GPP and LA (6.584 days) and nuggets with GPP and PE (3.927 days). The meat sample where all of the GPP, LA and PE were combined became unacceptable for high mesophilic growth after about 10 days. The calculated normalized percent difference between the shelf life of the sample with the most antimicrobials and that of the sample with the least antimicrobials demonstrates that, among all of the comparisons of the tested samples, we recorded ∆%>1, which means that all of the active compounds selected are effective in extending the food’s shelf life, but that differences among them were recorded. ∆%_2 and ∆%_3 , which refer to the efficacy of GPP double ∆%_1, which in turn refers to LA, thus suggesting that GPP is more effective than LA in extending the shelf life of chicken nuggets. ∆%_4 and ∆%_5, referring to the effects of PE on chicken meat, are both positive but much less so than ∆%_2 and ∆%_3, thus indicating that PE exerted slight effects when compared with LA and GPP. When combined, the three compounds promoted a very high normalized percent difference (∆%_6=1209.50), due to a recognized synergic effect among them. The ∆%_7 (853.77) is the normalized percentage increase of shelf life individually obtained by using LA, PE, and GPP, compared with shelf life of the control without any active compounds. Given that ∆%_7 simply added the effects that individual antimicrobials can have on shelf life, it is worth noting that it was lower than ∆%_6 because the antimicrobials can act synergistically in controlling meat detrimental phenomena.

Within the broader goals of sustainable food preservation, the paper is a valid example of the valorization of industrial residues. From a scientific point of view, further explorations can be made to deepen the research on the mechanisms mainly responsible for the recorded effectiveness. In any case, these results are very promising for the meat sector because they demonstrate that natural ingredients coming from by-products can substitute synthetic compounds for the preservation of fresh meat, with advantages for human health and environment. A proper cost–benefit analysis could support the subsequent industrial scalability.

## Figures and Tables

**Table 1:** Formulation of the batter used for the different chicken nuggets samples.

Ingredients	#A (g)	#B (g)	#C (g)
Sparkling water	95	95	95
Wheat flour 00	55	25	25
Pomace powder	-	30	30
Pomegranate extract	-	-	12

**Table 2:** Chicken nugget formulations for control and active samples.

Sample	Dipping in Lactic Acid	Batter	Breaded Coating
CTRL 1	no	#A	Breadcrumb
CTRL 2	yes	#A	Breadcrumb
SAMP_1	no	#B	GPP
SAMP_2	yes	#B	GPP
SAMP_3	no	#C	GPP
SAMP_4	yes	#C	GPP

**Table 3:** Antioxidant properties of GPP and PE samples.

Sample	EC50 (mg/mL) (DPPH)	Trolox Equivalents (FRAP)	Trolox Equivalents (CUPRAC)	Gallic Acid Equivalents (TPC)
GPP	0.365 ± 0.041 ^a^	0.024 ± 0.002 ^b^	0.049 ± 0.003 ^b^	0.045 ± 0.001 ^b^
PE	0.031 ± 0.001 ^b^	0.645 ± 0.028 ^a^	0.316 ± 0.003 ^a^	0.221 ± 0.001 ^a^

Data are presented as mean ± SD. Data in each column with different superscript letters are statistically different (*p* < 0.05). GPP = grape pomace powder; PE = pomegranate extract.

**Table 4:** Data regarding the microbial acceptability limit (MAL) calculated from the fitting procedure of sensory experimental data of tested chicken nuggets samples.

Sample	MESOF	MAL				
		PSY	LAB	ENTER	PSEUD	STAPH
CTRL_1	1.804 ± 0.299 ^d^	0.762 ± 0.164 ^e^	>13	1.8165 ± 0.163 ^a^	1.151 ± 0.207 ^c^	>13
CTRL_2	2.650 ± 0.210 ^d^	2.099 ± 0.162 ^d^	>13	2.2175 ± 0.233 ^a^	1.730 ± 0.171 ^c^	>13
SAMP_1	7.156 ± 0.349 ^c^	3.989 ± 0.335 ^c^	10.331 ± 0.481 ^b^	>13	2.905 ± 0.406 ^b^	>13
SAMP_2	7.924 ± 0.371 ^b,c^	6.780 ± 0.211 ^b^	13.029 ± 0.996 ^a^	>13	6.584 ± 0.225 ^a^	>13
SAMP_3	8.578 ± 0.470 ^b^	5.965 ± 0.474 ^b^	>13	>13	3.927 ± 0.698 ^b^	>13
SAMP_4	9.978 ± 0.541 ^a^	10.725 ± 0.388 ^a^	>13	>13	>13	>13

Data are presented as mean ± SD. Data in each column with different superscript letters are statistically different (*p* < 0.05). CTRL_1 = meat sample without any treatment; CTRL_2 = meat sample treated with lactic acid; Samp_1 = meat sample treated with grape pomace powder; Samp_2 = meat sample treated with grape pomace powder and lactic acid; Samp_3 = meat sample treated with grape pomace powder and pomegranate extract; Samp_4 = meat sample treated with grape pomace powder, lactic acid and pomegranate extract. PSY = psychrotrophic bacteria; LAB = lactic acid bacteria; ENTER = Enterobacteriaceae; MESOF = mesophilic bacteria; PSEUD = *Pseudomonas* spp.; STAPH = *Staphylococcus* spp.

**Table 5:** Data of sensory acceptability limit (SAL) calculated from the fitting procedure of the sensory experimental data of tested chicken nuggets samples.

Sample	SAL	
	QG Uncooked [Day]	QG Cooked [Day]
CTRL_1	7.337 ± 0.191 ^b^	7.778 ± 0.191 ^b^
CTRL_2	7.495 ± 0.218 ^b^	8.028 ± 0.243 ^b^
SAMP_1	10.351 ± 0.433 ^a^	13.703 ± 0.840 ^a^
SAMP_2	10.508 ± 0.533 ^a^	13.445 ± 0.784 ^a^
SAMP_3	11.545 ± 0.538 ^a^	>13
SAMP_4	11.180 ± 0.741 ^a^	>13

Data are presented as mean ± SD. Data in each column with different superscript letters are statistically different (*p* < 0.05). CTRL_1 = meat sample without any treatment; CTRL_2 = meat sample treated with lactic acid; Samp_1 = meat sample treated with grape pomace powder; Samp_2 = meat sample treated with grape pomace powder and lactic acid; Samp_3 = meat sample treated with grape pomace powder and pomegranate extract; Samp_4 = meat sample treated with grape pomace powder, lactic acid and pomegranate extract.

**Table 6:** Data of shelf life (day) calculated from the fitting procedure.

Sample	Shelf Life [Day]
CTRL_1	0.762 ± 0.164 ^e^
CTRL_2	1.730 ± 0.171 ^d,e^
SAMP_1	2.905 ± 0.406 ^c,d^
SAMP_2	6.584 ± 0.225 ^b^
SAMP_3	3.927 ± 0.698 ^c^
SAMP_4	9.978 ± 0.541 ^a^

Data are presented as mean ± SD. Data in each column with different superscript letters are statistically different (*p* < 0.05). CTRL_1 = meat sample without any treatment; CTRL_2 = meat sample treated with lactic acid; Samp_1 = meat sample treated with grape pomace powder; Samp_2 = meat sample treated with grape pomace powder and lactic acid; Samp_3 = meat sample treated with grape pomace powder and pomegranate extract; Samp_4 = meat sample treated with grape pomace powder, lactic acid and pomegranate extract.

## Data Availability

The original contributions presented in the study are included in the article, further inquiries can be directed at the corresponding author.
